# Anti-TNFα therapy in IBD alters brain activity reflecting visceral sensory function and cognitive-affective biases

**DOI:** 10.1371/journal.pone.0193542

**Published:** 2018-03-08

**Authors:** Marcus A. Gray, Che-yung Chao, Heidi M. Staudacher, Natasha A. Kolosky, Nicholas J. Talley, Gerald Holtmann

**Affiliations:** 1 Department of Gastroenterology & Hepatology, Princess Alexandra Hospital Brisbane, Queensland, Australia; 2 Translational Research Institute, Brisbane, Queensland, Australia; 3 Faculty of Health and Behavioral Science, University of Queensland, St. Lucia, Brisbane, Queensland, Australia; 4 Faculty of Medicine, University of Queensland, St. Lucia, Brisbane, Queensland, Australia; 5 Faculty of Health and Medicine, University of Newcastle, Newcastle, New South Wales, Australia; Universita Cattolica del Sacro Cuore Sede di Roma, ITALY

## Abstract

**Background:**

In inflammatory bowel disease (IBD), immune activation with increased circulating TNF-α is linked to the intensity of gastrointestinal symptoms and depression or anxiety. A central feature of depression is cognitive biases linked to negative attributions about self, the world and the future. We aimed to assess the effects of anti-TNFα therapy on the central processing of self-attribution biases and visceral afferent information in patients with Crohn’s disease.

**Methods:**

We examined 9 patients with Crohn’s disease (age 26.1±10.6. yrs, 5 female, 5 ileocolonic, 2 colonic and 2 ileal disease) during chronic anti-TNFα therapy (5 adalimumab, 4 infliximab). Patients were studied twice in randomized order before and after anti-TNFα administration. On each occasion patients underwent functional magnetic resonance imaging (fMRI) of the brain during a test of implicit attribution biases regarding sickness/health and undertook a standardized nutrient challenge.

**Results:**

Following anti-TNFα treatment, ratings of ‘fullness’ following nutrient challenge reduced compared to pre-treatment ratings (p<0.05). Reaction times revealed improved processing of self-related and positive health words, consistent with improved implicit sense of wellbeing that correlated with improvements in sensory function after treatment (r = 0.67, p<0.05). Treatment-associated improvements in implicit processing were mirrored by alterations of prefrontal, amygdala, posterior cingulate and visual regions. Between patients, the degree of functional amygdala change was additionally explained by individual differences in attention regulation and body awareness rankings.

**Conclusion:**

In patients with Crohn’s disease, anti-TNFα administration reduces visceral sensitivity and improves implicit cognitive-affective biases linked to alterations in limbic (amygdala) function.

## Introduction

Inflammatory bowel diseases (IBD), including Crohn’s disease and ulcerative colitis, are chronic intestinal diseases that result in a substantial disease burden [1] including an increased incidence of comorbid depression and anxiety [2]. Management of IBD however often neglects to adequately screen and treat depression and anxiety [3, 4]. Potent immunomodulation via long term inhibition of the natural cytokine, tumor necrosis factor alpha (TNFα), heals mucosal lesions in a significant proportion of patients with IBD [5, 6]. Both symptoms of depression and anxiety are more prevalent when IBD is active versus when in remission, however gastrointestinal and psychological symptoms persist in a substantial proportion of patients despite mucosal healing [7, 8].

Recently the relationship between peripheral inflammation and psychiatric symptoms including depression and anxiety has become an important focus within biological psychiatry. There is a growing recognition that peripheral inflammation induces coordinated “sickness behaviors” which appear largely identical to depressive symptoms [9, 10]. This has been demonstrated in neuroimaging studies by ourselves and others in which induced peripheral inflammation in healthy adults increases depressive symptoms and alters limbic and prefrontal activity within the brain [11–13]. Specific patterns of cognitive and affective change (or biases) are central to both the development and maintenance of depression[14]. These cognitive-affective biases alter attributions about the self, the world and the future[15]. Importantly, beyond simply identifying associations between depression and inflammation, research is now beginning to identify specific cognitive affective processes and their neural underpinnings which are altered during an inflammatory state, both in humans and experimental animals [16, 17].

A range of inflammatory markers in IBD provide sensitive measures of peripheral inflammation and have been strongly correlated with depression [18]. However, while providing sensitive measures of inflammation, blood borne inflammatory markers poorly localize at the site of inflammation. Faecal calprotectin, conversely, indexes neutrophil infiltration into gastro-intestinal (GI) mucosal tissue and is the noninvasive gold standard for indexing inflammation of the gastrointestinal mucosa in IBD, reflecting mucosal inflammation in Crohn’s Disease (CD) more accurately than C-reactive protein or clinical scores such as the Crohn’s Disease Activity Index (CDAI) [19, 20]. Treatment with anti-TNFα, a widely used therapy in patients with IBD[21], effectively modulates immune function in the gastrointestinal tract, and appears to reduce sickness behavior and depressive symptoms in IBD [22, 23], rheumatoid arthritis [24] and psoriasis [25].

The impact of targeted modulation of the immune system on cognitive-affective biases characteristic of depression in patients with inflammatory bowel disease has not previously been examined. We thus hypothesized that the modulation of the immune-system by administration of anti-TNFα alters interoceptive signaling (i.e. ongoing monitoring of visceral bodily state [26–28], changing activity within prefrontal and limbic circuits of the brain which underlies specific cognitive and affective processing characteristic of depression patients with IBD.

## Methods and materials

### Participants

After approval of the protocol by the national Human Research Ethics Committee (Metro South, HREC/12/QPAH/654) and the University of Queensland Human Research Ethics Committee, we recruited patients with Crohn’s disease who were receiving standard clinical treatment. These patients’ symptoms had been stabilized by regular anti-TNFα administration and had CDAI (Crohn’s Disease Activity Index) scores of <150 confirmed for at least 6 months. After providing full study information, we obtained informed consent from 11 patients who chose to participate in the research project. Patients were attending the Princess Alexandra Hospital Inflammatory Disease Outpatient Clinic and receiving either adalimumab every 2 weeks, or infliximab every 8 weeks, as per normal treatment schedules. Exclusion criteria included pregnancy, previous neurological disease or injury or contra-indication for Magnetic Resonance Imaging (MRI). One patient withdrew from the study before testing sessions were completed, and for 1 additional patient functional MRI (fMRI) data could not be used due to a technical problem, leaving a study sample of 9 participants (mean age = 26.1±10.6 years, 5 were female, 5 treated with adalimumab, 4 with infliximab). The mean time since diagnosis was 4.8 years, and on average patients had been treated with TNF-α for 27.7 months. 2 patients had small bowel disease, 2 had colonic disease, and 5 had both. 3 patients had peri-anal fistulas. No patients had undergone intestinal surgical interventions (Table 1). Interestingly, despite all patients reporting only minimal gastrointestinal symptoms and scoring below 150 on the CDAI, calprotectin levels reveal significant inflammation in 6/9 patients (adopting the conservative threshold published by D’Haens et al., 2012 of >250 μg/g [20]).

**Table 1:** Patient demographic and clinical information.

Age	Sex	Age at Diagnosis	Anatomical Location	Disease behavior	Growth Delay	Year since Diagnosis	Months since starting Anti-TNF	Generic anti-TNF drug	Anti-TNFα Dose	Additional immune suppressant	Pre-treatment fecal calprotectin μg/g	Post treatment Fecal calprotection μ g/g	IBDQ pre-treatment
31.9	F	A2	L2	B1+P	Na	3	24	adalimumab	40mg *	methotrexate	1100	530	131
25.9	F	A1b	L2	B2	No	12	33	infliximab	5mg/kg **	Nil	1100	500	196
19.2	M	A1b	L1+4a	B1	No	5	55	infliximab	5mg/kg **	Nil	680	98	178
20.1	F	A2	L3	P	Na	1.75	18	adalimumab	40mg *	Nil	1600	150	216
18.0	F	A1b	L3	B1	No	3	12	adalimumab	40mg *	mercaptopurine	44	27	185
24.3	M	A2	L3	B2+P	Na	5.5	36	adalimumab	40mg *	Nil	1300	1300	135
51.7	F	A3	L3	B3	Na	7	42	adalimumab	40mg *	azathioprine	19	36	182
25.9	M	A2	L3	B1	Na	4	22	infliximab	5mg/kg **	azathioprine	250	310	166
18.3	M	A1b	L1	B1+P	No	2	7	infliximab	5mg/kg **	mercaptopurine	20	20	183

Montreal classification system is used for participants ≥ 17 years of age at diagnosis, and the Paris classification system is used for participants <17 years at diagnosis.

Age at diagnosis—A1a: 0-10yrs, A1b: 10-17yrs, A2: 17–40 years, A3 > 40 years.

Anatomical distribution—L1: Distal 1/3 ileum ± limited cecal disease, L2: colonic, L3: ileocolonic, L4a: upper disease proximal to Ligament of Treitz, L4b: upper disease distal to.

Ligament of Treitz and proximal to distal 1/3 ileum.

Disease behavior—B1: Non-stricturing Non-penetrating, B2: Stricturing, B3: Penetrating, B2B3: both penetrating and structuring disease, either at different or same times, P: Peri-anal.

* 40mg of adalimumab was administered every 2 weeks.

** 5 mg/kg of infliximab was administered every 8 weeks.

## Procedure

In randomized order, each participant twice completed brain scanning assessments whilst undertaking an experimental task (Implicit Associations Test), and then underwent viscerosensory testing. Assessments occurred 48 hours before receiving anti-TNFα therapy, to coincide with trough plasma anti-TNFα levels, and again either 48 or 96 hours after administration of infliximab or adalimumab, to coincide with peak (t max) anti-TNFα plasma concentrations. On each testing day, fMRI scanning was performed at the Centre for Advanced Imaging (CAI) at the St. Lucia Campus of the University of Queensland. On the first day of testing, all participants completed validated psychometric questionnaires assessing 3 general domains; 1) interoceptive awareness (i.e., sensitivity to internal physiological sensations), 2) GI symptoms, and 3) anxiety and depression symptoms. Interoceptive awareness questionnaires included the Body Perception Questionnaire [BPQ[29]], and the Multidimensional Assessment of Interoceptive Awareness [MAIA[30]]. GI symptoms were assessed via the Inflammatory Bowel Disease Questionnaire [IBDQ[31]] and symptom ratings during the standardized nutrient challenge as described below [32]. Anxiety and depression were assessed via the Hospital Anxiety and Depression Scale [HADS[33]], the Beck Depression Inventory [BDI[15]] and the State-Trait Anxiety Inventory—Trait Version [STAI-T[34]]. Additionally, on each day of testing the state version of the STAI was completed.

### Implicit Associations Test

**Fig 1:**
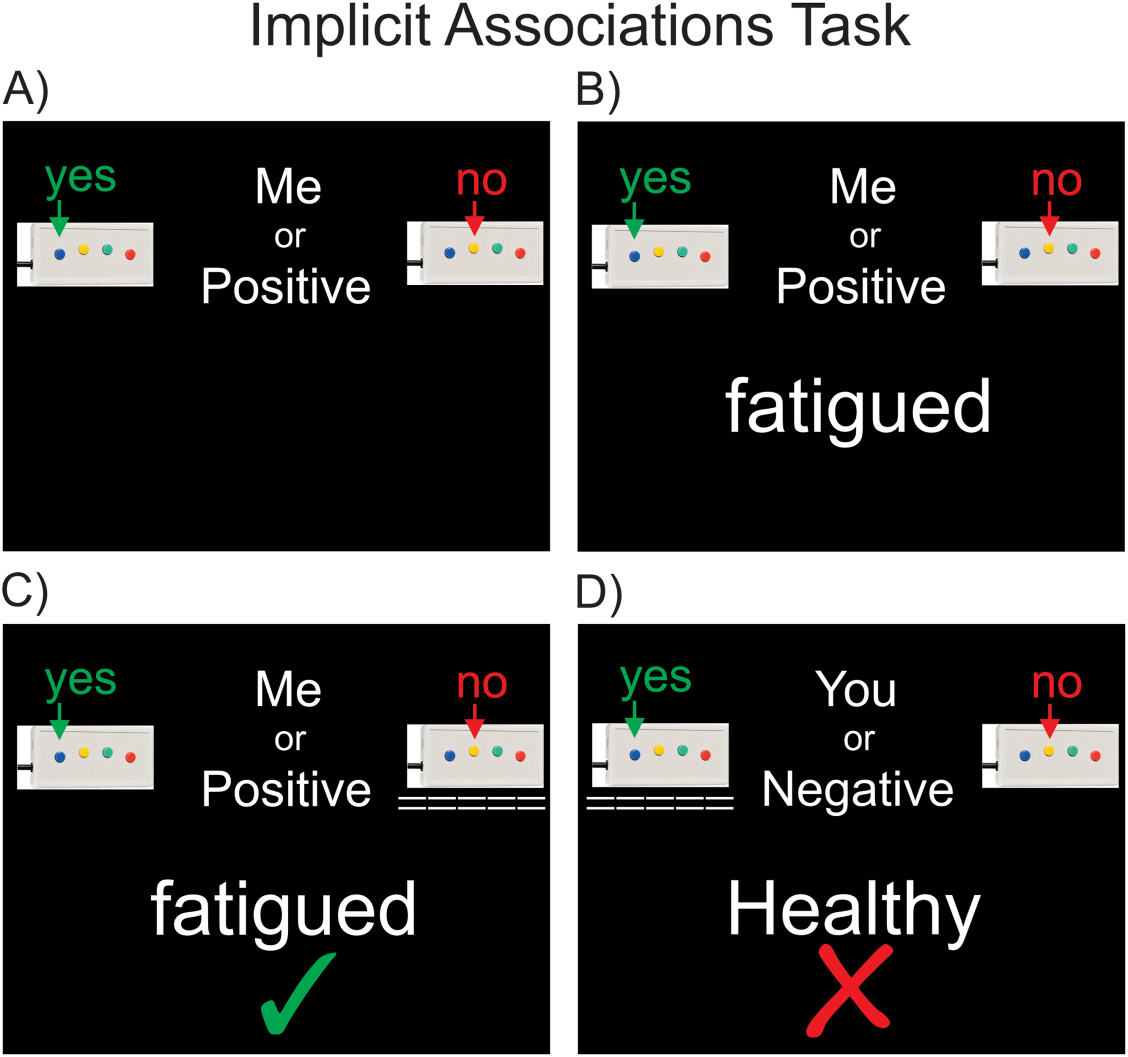
Implicit Associations Task—Experimental stimuli. A) Task blocks begin by identifying the classification condition. In this condition, participants must decide if the stimulus word is self-related or positively valenced (Me or Positive). The stimulus word is then displayed (B), which the participant then classifies (C). In this case the participant correctly chose that the stimulus word “fatigued” was not a “Me or Positive” word. Visual feedback of each choice is displayed under the yes or no button box (dashed lines), and feedback about the classification accuracy is displayed below the stimulus word, in this case with a green tick. D) In this example, the participant incorrectly chose that the stimulus word “healthy” was an other-related or negatively valenced word (You or Negative). Participants are reminded of which buttons of the 4 button response box they are holding indicate yes and no by the illustrations at the top left and right of each screen.

The Implicit Associations Test (IAT), first developed by Greenwald et al. (1998), allows investigation of how strongly one’s self-concept is associated to positive or negative attributes, compared to their association strength to others [35, 36]. The underlying logic is that highly associated categories (such as oneself and positive attributes) are processed more easily (i.e. faster) than less associated categories (for example, oneself and negative attributes). We modified this task by contrasting positive somatic and psychological attributes (happy, hopeful, energized, eager, valuable, clever, attractive, honorable, clear headed, innocent, strong and healthy) with negative attributes characteristic of illness and depression (depressed, fatigued, damaged, hopeless, worthless, stupid, ugly, pathetic, confused, guilty, weak, sick). In addition to positive and negative words, self-related and other-related words were also used (e.g. me, mine, my, you, them, they) (for further information on IAT word lists see supporting information S1 Text). Prior to the task, participants were asked to familiarize themselves with these wordlists. Participants were instructed to rapidly classify (yes/no) each stimulus word into four classification conditions, each of which combined two of the individual stimulus word categories. For example, was the stimulus word a “self-related or positive word?” (y/n), a “self-related or negative word?” (y/n), an “other-related or positive word?” (y/n), or an “other-related or negative word?” (y/n) (Fig 1). The order of the classification conditions was randomized, and the order of the stimulus words was also randomized within each classification block. It is important to note that the same stimulus words were presented during each classification condition; *only the classification category changed*, see Greenwald for a further description [35, 36]. Critically for this experiment, participants completed this task twice, at peak and trough levels of anti-TNFα, allowing a direct examination of how responses were influenced by inflammatory state. This effect was tested with a 2x2x2 repeated measures ANOVA to test for the influence of treatment (pre vs post), identity (self-related vs other-related) and valence (positive vs negative) on reaction times. This was followed by subsequent 2x2 ANOVA’s first split across levels of valence (positive vs negative) and then split across levels of identity (self versus other), where the effect of treatment on cognitive-affective bias is revealed in the interaction.

Prior to each scanning session, all participants were shown a scripted set of task instructions, and had the words from each stimulus category presented to them. They then completed a short practice task with experimenter guidance and feedback to ensure accurate task performance. During brain scanning, stimuli were projected onto a screen behind the magnet bore from a projector outside the scanner room and viewed via an angled mirror fitted to the MRI head coil. Responses were made via an MRI compatible fiber optic button box, with reaction times recorded by the stimulus presentation program.

### fMRI acquisition

Blood-oxygenation-level-dependent (BOLD) weighted echo-planar imaging (EPI) was acquired on a Siemens 3Tesla “Trio” MRI scanner. We acquired rapid multiband imaging with full brain coverage at a spatial resolution of 2.5mm isotropic voxels. See supporting information S2 Text for further sequence information. fMRI images were acquired in a single continuous run while participants completed the experimental task. On average, participants completed the experimental task in 11min 55s (±30.2s).

### fMRI preprocessing and analysis

The fMRI data were pre-processed and analyzed using Statistical Parametric Mapping 12 (SPM12) software and MATLAB (version 8.2 2013b). Raw images were realigned, screened for artifacts with Artrepair (version 4, Stanford University), and normalized via the segment routine, prior to writing at 2mm isotropic spatial resolution and smoothing with an 8mm FWHM (full width at half maximum) kernel. See supporting information S3 Text for further information.

First level modeling, performed at the single subject level, included regressors for each of the four classification conditions (me or positive, me or negative, you or positive, and you or negative), and their first order temporal derivatives to account for any simple decreases in attentiveness during the scanning session. Simply put, this allows the identification of neural regions engaged by each of the 4 task conditions. See supporting information S4 Text for further information. Group effects were then assessed via the standard SPM second level (hierarchical modeling) approach [37]. This second level model contained estimates (regressors) of the four classification conditions for each participant, during both the pre anti-TNF-α and post anti-TNF-α sessions. Specifically, a flexible factorial model was constructed in SPM12 with separate factors of subject, treatment and task. The second level design matrix estimated the main effects of treatment and task, and their interaction.

### Neural regions of interest (ROI’s)

Our expectation was that neural activity within the frontal lobes and limbic regions would likely underlie any behavioral biases during the IAT task. A number of previous brain imaging studies reveal that implicit biases during the IAT reflect neural responses within the prefrontal cortex and limbic / paralimbic system (in particular within the cingulate cortex and amygdala)[38–41]. In addition to exploring responses anywhere within the brain (with the appropriate family wise error controlled statistical threshold) we also adopted a focused Region of Interest (ROI) approach by restricting the search space to prefrontal and limbic regions. We constructed a combined mask of the entire prefrontal cortex, cingulate gyrus and amygdala using Wake Forest University PickAtlas (version 3.0.5, http://fmri.wfubmc.edu/software/pickatlas). All activation clusters were thresholded at a minimum cluster size of k>20 voxels. Reported findings were restricted to those above the family-wise error correction threshold for the combined regions of interest.

### Viscerosensory testing

After functional imaging was completed, all participants completed a standardized nutrient challenge in order to evaluate viscerosensory function. Participants rated their symptoms prior to consumption of a liquid meal (Resource plus, Nestle Healthcare, caloric density 1.5 kcal/ml: 5.5 g protein, 22.6 g carbohydrate, 4.5 g fat/100 ml). Symptoms (pain, fullness, nausea, burning and regurgitation) were assessed using a visual analogue scale (range 0-100mm) where “0” = no symptoms and “100” = very severe symptoms. Participants then consumed 200ml every five minutes up to a total of 600ml (or the maximum ingested volume if subjects did not tolerate 600 ml), and again rated their symptoms [42]. We have previously demonstrated that symptom severity at a cumulative volume of 600 ml is negatively correlated with the gastric volume threshold for discomfort measured with a barostat device [43]. Paired t-tests tested for treatment associated changes in subjective ratings. Finally, associations between treatment effects on IAT responses, neural activity underlying IAT responding, viscerosensory testing and psychometric measures were explored via Pearson’s pairwise correlations. All authors had access to the study data, and reviewed and approved the final manuscript.

## Results

### Viscerosensory testing

**Fig 2:**
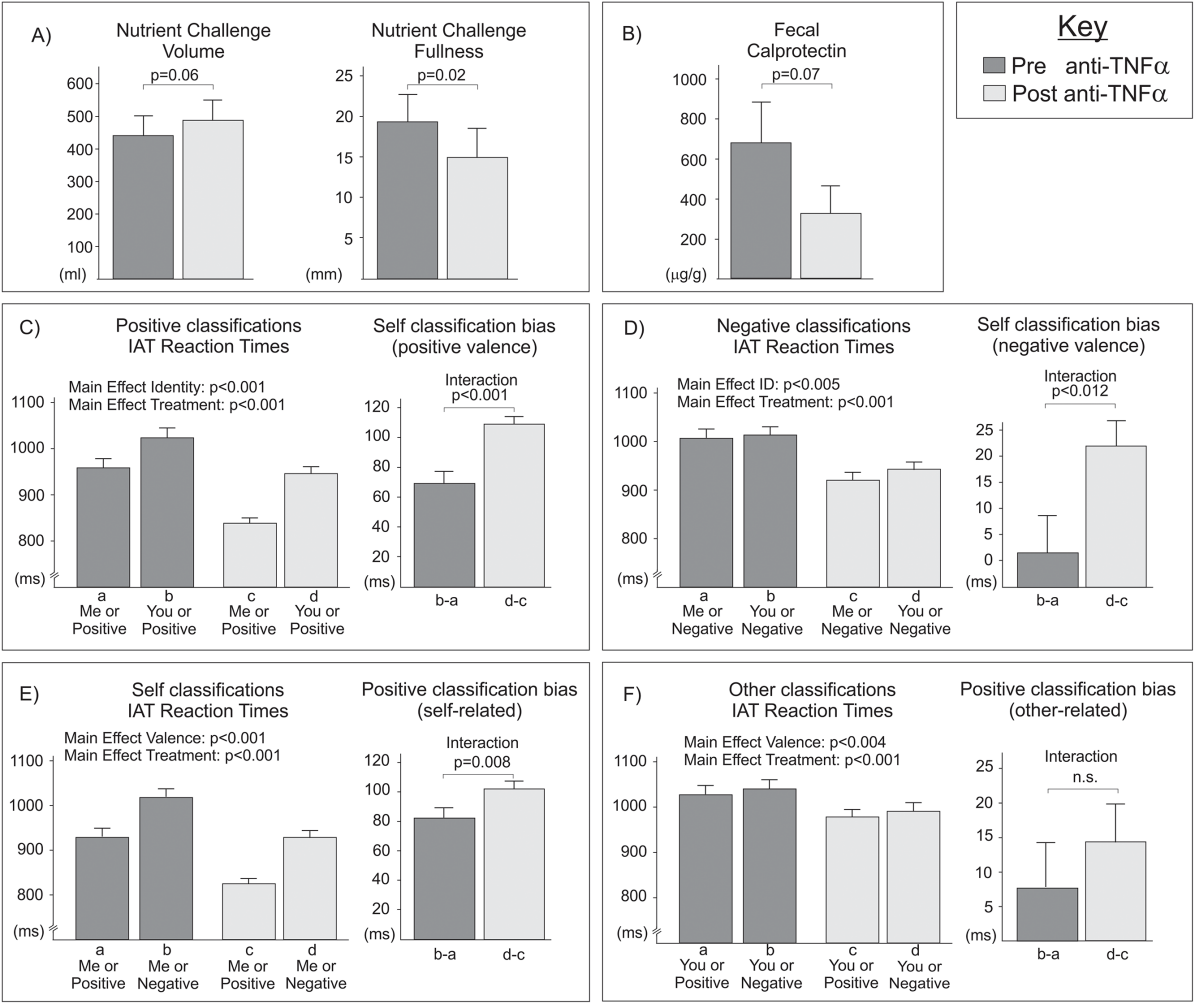
Influence of anti-TNFα on subjective symptoms and classification biases. A) The maximal tolerated volume of nutrient challenge tended to be higher after treatment (p = 0.06)(left); while subjective fullness was significantly reduced following anti-inflammatory therapy (p = 0.02) (right). Panels B-E present reaction times (left bar-graphs) underlying classification biases (right bar-graphs). Faster responses were made to self versus other related words (B-C), and to positive versus negative words (D-E). Treatment also reduced reaction times (B-E), and treatment x classification interactions revealed greater treatment effects for self-related and positive processing (B-D but not E). The significant three way interaction revealed anti-TNFα preferentially improved positively valenced self classification bias (B) over and above improvements in negatively valenced self classification bias (C), self-related positive classification bias (D) or other-related positive classification bias (E; no significant change).

The overall symptom response was significantly reduced (p<0.05) and there was a trend towards increased volume consumed after anti-TNFα administration (p = 0.06, Fig 2a).

### Calprotectin

Pairwise comparison of fecal calprotectin concentrations before and after treatment (paired t-test) did not reveal a significant decrease following anti-TNFα administration, although we did see a non-significant trend in this direction (p = 0.07, Fig 2b)

### Implicit Associations Test

Reaction time measures for identification of each classification category were filtered, removing responses which were less than 50ms, and trials where no behavioral response was made. Incorrect yes/no responses were excluded from analysis. We began by performing a 2x2x2 repeated measures ANOVA, to test for the influence of treatment (pre vs post), identity (self-related vs other-related) and valence (positive vs negative) on reaction times. We observed a significant 3 way interaction [F(1,191) = 9.364, p = 0.003]; anti-TNFα treatment produced greater increases in self-classification bias for positive words (Fig 2C) than comparable increases for negative words (Fig 2D), or increases in positive classification bias for self-related words (Fig 2E). To fully characterize this 3-way interaction, we performed subsequent ANOVA’s first split across levels of valence (positive vs negative) and then split across levels of identity (self versus other).

We first investigated positively valenced classifications only, via a 2x2 repeated measures ANOVA with the factors of identity (self v other) and anti-inflammatory treatment (pre v post) (Fig 2C) We observed main effects of treatment [F(1,254) = 93.47, p<0.001], and identity [F(1,254) = 346.4.3, p<0.001], and a significant interaction [F(1,254) = 22.4, p<0.001]. Simply put, reaction times were faster following treatment, and self-related were faster than other-related classifications. The interaction reflects a greater facilitation of self-related (positive) classifications following treatment.

An identical pattern of results was seen when considering only negatively valenced classifications (Fig 2D). We observed a main effect of treatment [F(1,246) = 86.0, p<0.001], a main effect of identity [F(1,246) = 15.9, p<0.001], and an interaction of treatment and identity [F(1,246) = 5.4, p = 0.021] Again, reaction times were faster following treatment, self-related were faster than other-related classifications and treatment facilitated self-related (negative) classifications.

Finally, we examined our results separately for self-related and other-related judgments. When considering self-related judgments (Fig 2E), we observed significant main effects of treatment [F(1,269) = 110.8, p<0.001] and valence [F(1,269) = 352.7, p<0.001], and a significant interaction [F(1,269) = 6.6, p = 0.011], such that treatment facilitated positive more than negative (self-related) classifications. However, when considering other-related judgments (Fig 2F), we again observed main effects for treatment [F(1,233) = 63.6, p<0.001] and valence [F(1,233) = 5.8, p = 0.017], but no treatment by valence interaction. Treatment effects therefore did not differ between positive and negative (other-related) classifications.

### Neural correlates

**Fig 3:**
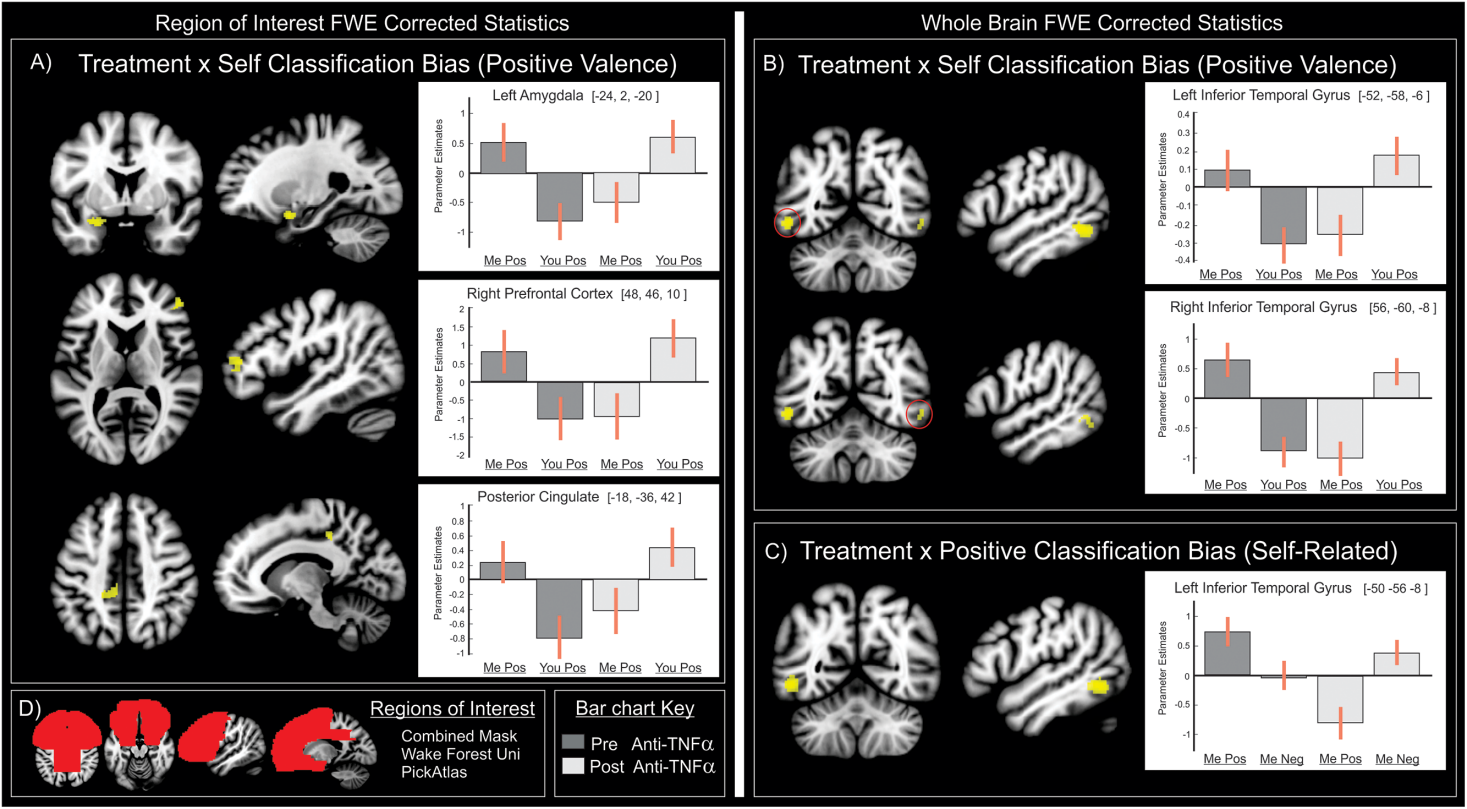
Neural responses underlying anti-inflammatory influences on classification biases. A) The predominant behavioral effect of anti-TNFα on increasing positively valenced self-classification bias directly reflected altered neural function within the left amygdala, right prefrontal cortex and left posterior cingulate. B) These effects were also seen outside our regions of interest, bilaterally in the inferior temporal gyrus. C) Responses within the inferior temporal gyrus also reflected behavioral increases in self-related positive classification bias. D) Region of interest inclusively masked entire frontal lobes, cingulate cortex and amygdala. Bar charts of parameter estimates are colored dark grey before and light grey after anti-TNFα treatment.

To identify the neural activity underlying anti-TNFα’s influence on cognitive-affective biases during the IAT, we matched the analysis of neural responses and behavioral responses; that is we analyzed first across levels of valence (positive vs negative) and then across levels of identity (self vs other). Neural responses during positively valenced classifications were examined within our a priori regions of interest (the entire frontal lobes, cingulate cortex and amygdala, Fig 3d). As with behavioral findings, we observed a significant influence of treatment on neural responses underlying self-related classification biases. Specifically, regional interactions were seen within the central nucleus of the left amygdala, within the pars triangularis region of the inferior frontal gyrus, and within the left posterior cingulate cortex (Fig 3a left and Table 2). Bar charts of parameter estimates reveal that prior to anti-inflammatory treatment, self-classifications activated these regions more than other classifications. Following treatment this pattern was reversed; self-related classifications evoked less regional activation than other related classifications. Examination of treatment effects on self-related classification biases with negative stimuli revealed no significant interactions in neural activity. Likewise, within our regions of interest, treatment effects on positive classification biases (for either self-related or other related classifications) were not associated with any significant alterations in neural response.

**Table 2:** Neural correlates of the interaction of anti-TNFα treatment with classification condition.

Region	L/R	(X Y Z) MNI	ClusterSize k	t-score	Region of Interest FWE p-value	Whole brain FWE p-value
**Self Classification Bias (positive valence)**						
Amygdala	L	-24, 2, -20	86	5.56	0.008	ns
Prefrontal Cortex	R	48, 46, 10	84	5.09	0.009	ns
Posterior Cingulate	L	-18, -36, 42	80	5.01	0.011	ns
Inferior Temporal Gyrus	L	-52, -58, -6	131	8.12	-	< 0.001
	R	56, -60, -8	30	5.79	-	0.008
**Positive Classification Bias (self-related)**						
Inferior Temporal Gyrus	L	-50, -56, -8	125	6.14	-	0.002

Family Wise Error (FWE) statistics are reported for the whole brain, and for activation clusters (k>20) within our regions of interest. XYZ MNI: the spatial distance in millimeters in the x, y and z directions from the origin in standardized Montreal Neurological Institute space.

For completeness, we also examined neural responses outside our regions of interest, while correcting for Family Wise Error across the entire brain. As above, we observed significant effects of treatment on self-related classification biases with positively valenced stimuli only (Fig 3B and Table 2). These interactions were seen within the posterior inferior temporal gyrus bilaterally. Within this region of the left hemisphere we additionally saw a significant effect of treatment on positive classification biases for self-related classifications but not for other-related classifications (Fig 3C and Table 2).

### Associations between classification biases, neural responses and psychometrics

**Fig 4:**
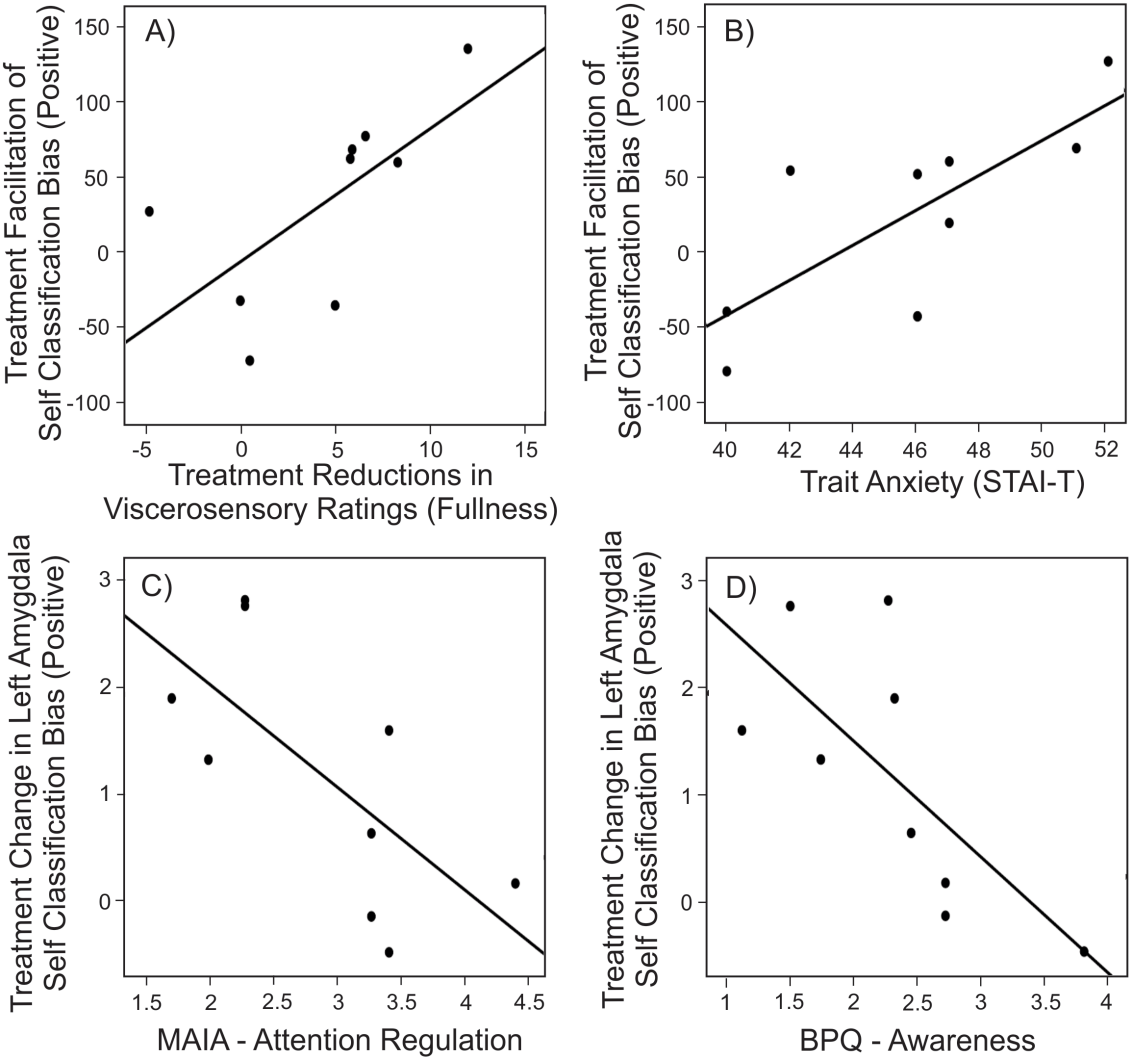
Associations between viscerosensory, behavioral neural and psychometric measures. Across participants, increased facilitation of positive self-classification biases were seen in participants with A) larger reductions in viscerosensory (fullness) ratings and B) higher levels of trait anxiety. Treatment associated alterations in left amygdala were largest in participants with C) reduced ability to regulate attention and D) reduced levels of awareness.

As an additional exploratory analysis, we tested for associations between our biological, behavioral, neural and psychometric measures. Importantly, we did not observe significant associations with disease state (indexed via pretreatment IBDQ score) or GI inflammation (indexed via fecal calprotectin concentrations and calprotectin change scores). These findings are presented without correction for multiple comparisons, to avoid missing small effects in an exploratory analysis, although the reader should be aware increases the potential for false positive findings. Four significant associations were observed. Across participants, reduced fullness ratings (after treatment) reflected facilitation in (positive) self-classification biases (Pearson’s r = 0.67, p<0.05). This indicates that across participants, the degree to which treatment facilitated self-related positive classifications (Fig 2B) reflected the degree to which treatment reduced symptoms of unpleasant fullness during the subsequent nutrient challenge provocation (Fig 4A). Improved self-related positive classification speed also reflected trait anxiety ratings taken during the first testing session [Pearson’s r = 0.77, p = 0.02] (Fig 4B). This suggests that inflammation associated interference in classifying positive self-related classifications was greatest in participants with higher trait anxiety ratings. We also observed that individual alterations in left amygdala function underlying positive self-related classification biases (Fig 3C) were reflected in psychometric measures of attention and awareness. Specifically, the degree to which treatment altered left amygdala responses reflected the attention regulation subscale of the MAIA (multi-dimensional assessment of interoceptive awareness) [Pearson’s r = -0.69, p = 0.04]. These changes in left amygdala response also reflected the awareness subscale of the Body Perception Questionnaire (BPQ) [Pearson’s r = -0.71, p = 0.03]. This suggests that interference in amygdala function underlying positive self classification biases were reduced in participants’ with greater attention regulation capacity (Fig 4C) and body awareness (Fig 4D).

## Discussion

The key findings of this study are: firstly, after administration of anti-TNFα the sensory response to a standardized (visceral) nutrient stimulus was significantly reduced compared with baseline (pre anti-TNFα). Secondly, anti-TNFα treatment was associated with increased self-related positive classification biases, and these increases were correlated with objective improvements in sensory function after a nutrient stimulus. Thirdly, improvements in classification bias after anti-TNFα reflected alterations in neural responses within the left amygdala, right prefrontal cortex, posterior cingulate cortex and primary visual regions. These findings are the first to report improvements in cognitive-affective processing in response to anti-inflammatory treatment in IBD patients. Cognitive-affective biases are central in the development and maintenance of depression [15]. Our novel findings of immune mediated alterations in the neural activity underlying cognitive-affective processing provides indirect support for the involvement of inflammatory cytokines like TNFα in depressive symptoms frequently observed in patients [7] with inflammatory gastrointestinal disorders [44, 45].

Our results demonstrate that anti-TNFα administration reduces overall symptoms after a standard nutrient challenge. Following treatment, the sensations of fullness evoked by the nutrient challenge were significantly reduced. Treatment also altered cognitive-affective biases, and their neural underpinnings. These effects can’t simply reflect the direct action of biological anti-TNFα drugs in the brain, as these monoclonal antibodies do not cross the blood brain barrier. Importantly however, our behavioral and neural findings were not associated with changes in fecal calprotectin, suggesting they did not directly follow from reduced GI wall inflammation. Recently, Hess et al reported that neural activity evoked by abdominal compression in patients with Crohn’s disease was attenuated one day and 28 days after anti-TNFα treatment [46], an effect, they note, which appeared too rapidly to depend on healing of the GI mucosa. Instead, reduced nociception at the dorsal root ganglia (for example via anti-TNFα inhibition of CX3CL1 / fractalkine [47]) may represent the underlying mechanism. A spinal transduction mechanism underlying rapid (24 hour) anti-nociceptive effects following anti-TNFα in IBD would also be consistent with the rapid reduction of pain reported in patients with rheumatoid arthritis [48]. Disturbed visceral-sensory mechanisms including the abnormal central processing of afferents have clearly been demonstrated to play an important role in the manifestation IBD and functional GI symptoms[46, 49–51]. The findings we present here are the first report of rapid reductions in unpleasant visceral symptoms following anti-TNFα treatment which directly reflect changes in cognition and alterations in limbic function.

Importantly, our findings demonstrate altered cognitive-affective processing following anti-TNFα treatment. A large number of social psychology studies have observed that healthy adults typically hold positive views of their own traits and behaviors which can be demonstrated via self-positivity biases in behavioral responses. Conversely, depression is characterized by less positive views of the self and cognitive slowing[14, 15, 52–54] and has been associated with reduced self-positivity biases[55, 56]. During the Implicit Associations Task, our participants clearly exhibited faster identification of both positively valenced and self-related words (i.e. positive classification bias and self-classification bias) soon after anti-TNF administration. Anti-TNFα treatment improved classification speeds generally, consistent with a general improvement in cognitive function. This supports important recent findings that anti-TNFα treatment can mitigate depressive symptoms in IBD[57] and additionally improve cognitive deficits in major depressive disorder[58]. Critically, we also observed that anti-TNFα treatment specifically improved self-positive classification biases, consistent with an improved implicit sense of wellbeing. The increasing use of patient reported outcome measures sensitive to depressive and anxious symptoms reflects the importance of psychological state to effective treatment [59, 60].

In our results, the largest changes in cognitive-affective processing efficiency were seen in participants with higher anxiety. Additionally, alterations of amygdala function which underpinned these behavioral changes were largest in participants with lower awareness and attention regulation scores. This may suggest a constellation of vulnerability, whereby frequently anxious individuals with reduced awareness and regulatory capacity are more influenced by gastrointestinal inflammation, and its treatment via anti-TNFα. Conversely, the fact that the viscerosensory and cognitive-affective consequences of inflammation also reflected measures of awareness and attention regulation may suggest that increasing these capacities in patients may help ameliorate symptoms Conclusions on these associations with psychometric measures observed in our small study should remain tentative however until confirmed in larger independent studies.

Our findings suggest that cognitive-affective processing central to anxious and depressive symptoms is altered by anti-TNFα administration. This represents the first demonstration in humans of the modulation of visceral sensory function, cognitive-affective processing and underlying neural activity by an anti-inflammatory agent. This suggests potentially beneficial effects on psychiatric symptoms such as depression and anxiety, comorbid with IBD. It was unexpected however that these effects were not directly linked to GI inflammation via changes in fecal calprotectin. Limitations of this study include our small sample size, and the fact that we did not directly characterize circulating cytokine or drug levels. As they stand, our findings suggest that immune activation is not only associated with visceral hyperalgesia but potentially plays a role in the manifestation of comorbid anxiety and depression. The evaluation of endoscopy score and blood borne inflammatory markers such as C-reactive protein and interlukin concentrations would provide a more informed characterization of disease state, and significantly advance our understanding of the broader potential impacts of anti-TNFα [61].

In summary, our findings demonstrate that in patients with Crohn’s disease, anti-TNFα administration reduces visceral sensitivity and improves implicit measures of cognition consistent with an improved sense of wellbeing. These cognitive changes are linked to alterations in prefrontal and limbic and paralimbic function following anti-TNFα. The data suggest that immune activation is associated with alterations of Central Nervous System (CNS) function that may play a key role for the extra-intestinal (neuropsychiatric) comorbidities in patients with inflammatory bowel disorders.

## Supporting information

S1 Text Implicit Associations Test word lists. This is a word document containing additional information about the Implicit Associations Test word lists.
(DOCX)

S2 Text fMRI acquisition. This is a word document containing additional information about the fMRI acquisition parameters.
(DOCX)

S3 Text fMRI preprocessing. This is a word document containing additional information about the fMRI preprocessing procedure.
(DOCX)

S4 Text fMRI 1st level processing. This is a word document containing additional information about the 1^st^ level modeling procedure.
(DOCX)
