# Supplementary material for: Anti-TNFα therapy in IBD alters brain activity reflecting visceral sensory function and cognitive-affective biases
Source: PLoS One. 2018 Mar 8;13(3):e0193542. doi: 10.1371/journal.pone.0193542 (PMC5843226; doi:10.1371/journal.pone.0193542)
Supplement: S1 Text — This is a word document containing additional information about the Implicit Associations Test word lists. (DOCX) [file pone.0193542.s001.docx]

**Supporting Information**

**S1 Text. Implicit Associations Test word lists.**

We carefully matched each category for word length, degree of positivity or negativity, degree of arousal and frequency of use in everyday language using normative lists for valence (intrinsic degree of attractiveness or averseness), arousal[1] and frequency of useage[2]. As designed, positive and negative words differed according to normative ratings of valence [F(1,21)=471, p<0.001] and arousal [F(1,21)=6.8, p<0.016], but did not differ in frequency of use in written language [F(1,21)=0.05, p=0.84] or length [F(1,21)=0.51,p=0.48].

The IAT task was programed in Matlab 2012b (The Mathworks, Inc., Natick, MA) using the Cogent 2000 toolbox (version 1.32, Wellcome Dept. of Imaging Neuroscience, London, UK). Classification categories were completed in 12 blocks (each classification category repeated 3 times) presented in a pseudo-randomized order. During each block four words from each stimulus category were presented sequentially, in a randomized order. Each block beginning was identified (“get ready” displayed for 500ms, fixation cross displayed for 500ms) followed by the classification condition required (Figure 1A). Pictures of the response buttons representing yes and no classifications remained continuously on the screen during each block. The first stimulus word was presented after 800ms, and participants were allowed up to 2400ms to respond, after which the stimulus word disappeared and the phrase “Too slow” was displayed. If participants did respond in time a green tick or a red cross was displayed below the stimulus word for the remaining response window plus an additional 800ms (Figure 1B-D). To improve independent signal estimation, each block ended with an additional 6 to 8 seconds of passive viewing (blank screen for 1000ms, fixation cross for 4000-6000ms, blank screen for 1000ms).

**References**

1. Warriner, A.B., Kuperman, V., and Brysbaert, M. (2013). Norms of valence, arousal, and dominance for 13,915 English lemmas. Behav Res Methods *45*, 1191-1207.

2. Hermit, D. (2016). Invoke IT Limited Frequency Word Lists. p. Frequency Word Lists
