# Supplementary material for: Anti-TNFα therapy in IBD alters brain activity reflecting visceral sensory function and cognitive-affective biases
Source: PLoS One. 2018 Mar 8;13(3):e0193542. doi: 10.1371/journal.pone.0193542 (PMC5843226; doi:10.1371/journal.pone.0193542)
Supplement: S2 Text — This is a word document containing additional information about the fMRI acquisition parameters. (DOCX) [file pone.0193542.s002.docx]

**Supporting Information**

**S2 Text. fMRI acquisition**

fMRI data was acquired with a 32-channel head coil on a Siemens 3T Trio MRI scanner (software version VB17A). We acquired data with an in plane spatial resolution of 2.5mm x 2.5mm. 36 slices were acquired ( 2.5mm thickness + 30% gap between slices), tilted approximately 30^0^ to the Anterior Commissure – Posterior Commissure axis in the sagittal plane, providing full brain coverage. We utilized a Work In Progress (WIP) sequence provided by Siemens to implement multiband imaging (acceleration factor of 4), resulting in a very rapid acquisition (full brain volume acquired TR= 540ms, TE=28ms, flip angle = 40^0^, field of view 1380x1380, acquisition matrix 92x92x36). The WIP sequence for rapid multi-band fMRI scanning provided by Siemens developers was named “Slice Accelerated BOLD EPI Blipped CAIPIRINHA Based FOV Shifting”.

In addition to fMRI data, a high resolution T-1 weighted image (full brain coverage) was also acquired for each participant using a 3D magnetization prepared rapid gradient recalled inversion recovery sequence (3D-MP2RAGE) (TR=4000ms; TE=2.89ms; 1mm3 isotropic voxels, 192 slices, acquisition matrix 240 x 256 x 192).
