# Supplementary material for: Anti-TNFα therapy in IBD alters brain activity reflecting visceral sensory function and cognitive-affective biases
Source: PLoS One. 2018 Mar 8;13(3):e0193542. doi: 10.1371/journal.pone.0193542 (PMC5843226; doi:10.1371/journal.pone.0193542)
Supplement: S3 Text — This is a word document containing additional information about the fMRI preprocessing procedure. (DOCX) [file pone.0193542.s003.docx]

**Supporting Information**

**S3 Text. fMRI preprocessing**

The fMRI data were pre-processed and analyzed using Statistical Parametric Mapping 12 (SPM12) software (version 6225-6685, Wellcome Department of Imaging Neuroscience, London, United Kingdom) and with MATLAB (Version 2012b-R2014b) for Windows; The Mathworks Inc., Natick, MA). After DICOM (Digital Imaging and Communications in Medicine) to NIFTI (Neuroimaging Informatics Technology Initiative) file format conversion, and within session realignment in SPM12, ArtRepair was used to screen and repair any artifacts at both the slice and volume level (ArtRepair, version 4, Stanford University). Normalization parameters were then generated via the SPM segment routine[37], applied to each functional volume at a spatial resolution of 2mm3, followed by smoothing with an 8mm FWHM Gaussian smoothing kernel in SPM12.
