# Supplementary material for: Anti-TNFα therapy in IBD alters brain activity reflecting visceral sensory function and cognitive-affective biases
Source: PLoS One. 2018 Mar 8;13(3):e0193542. doi: 10.1371/journal.pone.0193542 (PMC5843226; doi:10.1371/journal.pone.0193542)
Supplement: S4 Text — This is a word document containing additional information about the 1st level modeling procedure. (DOCX) [file pone.0193542.s004.docx]

**Supporting Information**

**S4 Text. fMRI 1st level processing**

Realignment parameters were included in first level design matrices to reduce any residual movement related artifacts. To ensure the highest quality fMRI data, additional CSF regressors (from bilateral ventricles and from outside the brain) were also added to the design matrix as regressors of no interest (although direct comparison revealed this additional artefact removal procedure had a negligible influence on the estimates of neural function).
